# Insights into the equilibrium structure and translocation mechanism of TP1, a spontaneous membrane-translocating peptide

**DOI:** 10.1038/s41598-022-23631-w

**Published:** 2022-11-18

**Authors:** Diego Muñoz-Gacitúa, Fanny Guzman, Boris Weiss-López

**Affiliations:** 1 Laboratorio de Fisicoquímica Molecular, Facultad de Ciencias, Universidad de Chile, Santiago, Chile; 2 Núcleo Biotecnología Curauma, Pontificia Universidad Católica de Valparaíso, 2460355 Valparaíso, Chile

**Keywords:** Peptides, Membrane structure and assembly, Computational models

## Abstract

Crossing the cellular membrane is one of the main barriers during drug discovery; many potential drugs are rejected for their inability to integrate into the intracell fluid. Although many solutions have been proposed to overcome this barrier, arguably the most promising solution is the use of cell-penetrating peptides. Recently, an array of hydrophobic penetrating peptides was discovered via high throughput screening which proved to be able to cross the membrane passively, and although these peptides proved to be effective at penetrating the cell, the details behind the underlying mechanism of this process remain unknown. In this study, we developed a method to find the equilibrium structure at the transmembrane domain of TP1, a hydrophobic penetrating peptide. In this method, we selectively deuterium-label amino acids in the peptidic chain, and employ results of $^2$H-NMR spectroscopy to find a molecular dynamics simulation of the peptide that reproduces the experimental results. Effectively finding the equilibrium orientation and dynamics of the peptide in the membrane. We employed this equilibrium structure to simulate the entire translocation mechanism and found that after the peptide reaches its equilibrium structure, it must undergo a two-step mechanism in order to completely translocate the membrane, each step involving the flip-flop of each arginine residue in the peptide. This leads us to conclude that the RLLR motif is essential for the translocating activity of the peptide.

## Introduction

Cells are competent at preventing ingress of foreign molecules, peptides and genetic material without a specific internalization mechanism. Due to this reason, one of the most restrictive factors during drug discovery is the inability of a potential drug to cross the cell membrane^1^; the current landscape dictates that a potential drug that is too polar will not be able to cross the cell membrane, and should be rejected^2,3^. For this reason, breaking this barrier would expand tremendously the universe of druggable compounds.

One of the most promising attempts to overcome the cell membrane as a barrier is the use of cell penetrating peptides (CPPs). These are peptides that are designed to cross the cell membrane, which can be attached to a cargo to assist its delivery to the cytosol. Since their discovery^4^, hundreds of CPPs have been proposed, many of them having been successfully tested in vivo^5,6^. Although it is essential knowledge for the development of peptide-based delivery systems, the internalization mechanism of CPPs remains an unsolved issue. This is because the specific mechanism not only depends on the physical–chemical properties, length^7^ and concentration^8,9^ of the peptide itself, but also on the properties of the cargo^10,11^. Many different mechanisms have been proposed and studied for CPPs in different conditions, such as endocytosis^12^, pore formation^13,14^, inverted micelles^15^, adaptive direct translocation^16^, among others^17,18^. Due to it having a great, but not determinant, influence on the translocation mechanism, one of the most common ways to classify CPPs is via their physical–chemical properties^19^, generating three categories: cationic, amphiphatic and hydrophobic. Due to the negative surface charge of the cell membrane, anionic CPPs are very limited^20^. Given that the first CPPs discovered featured a great number of arginines and lysines in their sequences^21,22^, most of the research regarding CPPs has been focused on the cationic category. On these highly cationic CPPs, most evidence points towards some type of endocytosis as the main mechanism^12,23,24^, with additional pathways when endocytosis is not possible^25^.

The main issue with this mechanism is that, albeit the CPP can effectively cross the cell membrane, it remains inside an endocyte, lowering considerably its bioavailability^26–30^. Many solutions have been proposed to improve endosomal escape to improve bioavailability of the cargo^31^, but a different approach has been redefining the way CPPs are designed in order to bypass completely endocytosis as the internalization mechanism^32,33^. This approach is the use of spontaneous membrane-translocating peptides^34–36^, which due to the requirement to have a relatively low polarity in order to translocate the membrane without mediation of an endocyte, are usually hydrophobic in nature.

Differently from cationic CPPs, which are commonly developed from naturally occurring proteins^4^, these hydrophobic CPPs are usually artificial in origin. Marks et al. developed a method in which a peptide library is screened through a synthetic lipid bilayer to identify spontaneous membrane translocating peptides^37^; the translocating peptide TP1 (Translocating peptide 1, sequence PLILLRLLRGQFC) emerged from this process, which has proven not only of being able to deliver polar cargo through synthetic lipid bilayers and in vitro cells membrane alike, but also being able to cross cell barriers in living animals and deliver cargo^34^.

As TP1 is able to translocate cells and synthetic vesicles alike, it can be concluded that the transport mechanism is passive in nature. In a later publication, Fuselier and Wimbley^35^ describe a common motif among the CPPs they found, LRLLR, which they found induces peptide translocation chaperoned by a lipid flip-flop mechanism. This finding goes in line with the strong interaction found between arginine and phosphate by many researchers^38–40^.

In this work, we aim to uncover the equilibrium structure and translocation mechanism of this peptide to understand the role of specific moieties of the CPP in the translocation process. In order to deduce the equilibrium structure, we used $^2$H-NMR spectroscopy, where deuterium-labeled molecules in an anisotropic media produce a split signal in which the magnitude of the splitting (called *quadrupolar splitting*) depends on the order parameter of the deuterium-labeled C–D bonds in the fragment, which in turn, depends on orientation and dynamics of the same fragment. In this way, by synthesizing multiples copies of the TP1 peptide with deuterium-labeled amino acids in different fragments and inserting these into an SDS-based (sodium dodecanoyl sulfate) synthetic bilayer sample^41^, we can obtain a set of order parameters belonging to each of the deuterium-labeled amino acids. We used this information, along with a calibrated molecular dynamics (MD) simulation model^42^, to calculate an equilibrium dynamic structure for the TP1 peptide inserted in the transmembrane domain.

Furthermore, with this equilibrium structure, we calibrated an MD simulation of the TP1 peptide translocating the same SDS-based synthetic bilayer, which we employed to propose a detailed mechanism, with a potential of mean force (PMF) profile included.

## Results and discussion

### Equilibrium structure of the TP1 peptide

**Figure 1:**
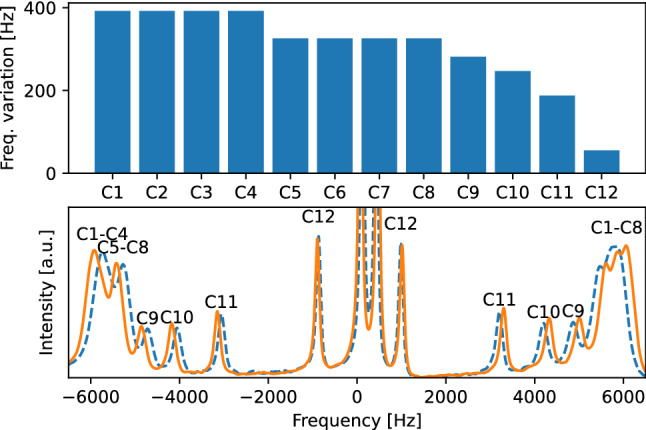
$^2$H-NMR spectra of SDS-based membrane mimetic enriched with SDS-d$_{25}$. Lower plot shows a comparison between samples with TP1 peptide (orange continuous line) and without it(blue dashed line). Upper plot show the variation in the quadrupolar splitting induced by the presence of TP1 peptide in the sample.

Before synthesizing any deuterated variations of the TP1 peptide, we performed a preliminary test with non-deuterated TP1 to confirm if the penetrating peptide is able to introduce itself into the SDS-based membrane mimetic. This test was performed by comparing the quadrupolar splittings in a $^2$H-NMR spectrum of an SDS-based membrane mimetic enriched with deuterated SDS (SDS-d$_{25}$) with another one also containing TP1 ($17\,\hbox {mg\,g}^{-1}$) peptide, see Fig. 1.

This SDS-based membrane mimetic was chosen due to its ability to generate oriented anisotropic liquid crystals, which is a requirement to observe quadrupolar splitting in $^2$H-NMR. Albeit the composition of this mimetic lacks phospholipids, it has been proven that SDS-based crystals are largely capable of mimicking interactions of transmembrane domains of proteins^43^.

Given that the quadrupolar splitting depends on the order parameter of the deuterium labeled fragment, and therefore on its orientational dynamics, the difference in quadrupolar splitting of the aliphatic tails induced by the addition of TP1 peptide can only mean that this peptide is at least heavily interacting with the bilayer surface of the SDS-based membrane mimetic. Also, considering that the effect is more notable on the quadrupolar splittings assigned to the fragments closer to the interface, we expect to find the peptide being hosted in this area at equilibrium.

Having confirmed that TP1 peptide strongly interacts with the bilayer of SDS-based membrane mimetic, we proceeded to synthesize the deuterated variations of the TP1 peptide for further $^2$H-NMR studies. Five variations were synthesized, four of them with deuterium labels in one of its leucines fragments, and a fifth one with deuterium labels in all five leucines fragments. Labeled leucines were triple deuterated in one of their $\delta$-carbon position, completely labeling one of two methyl groups.

**Figure 2:**
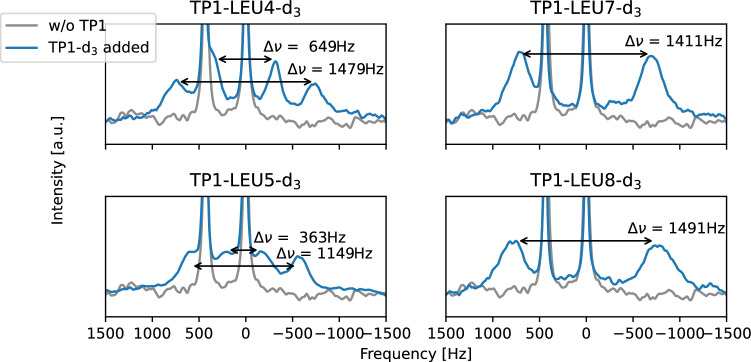
$^2$H-NMR spectra of TP1 peptide immersed in a SDS-based membrane mimetic. A different leucine is deuterium-labeled in each spectrum.

Figure 2 shows the $^2$H-NMR spectra obtained from these four deuterium-labeled TP1 peptides embedded into SDS-based membrane mimetics. Fragments Leu-7 and Leu-8 display a single quadrupolar splitting each, meaning that these fragments have a single most probable average orientation. Fragments Leu-4 and Leu-5, which have two quadrupolar splittings each, meaning that these fragments can be found in two possible average orientations and these orientations are stable enough not to alternate during the course of a single acquisition time in the $^2$H-NMR experiment (760 ms).

**Figure 3:**
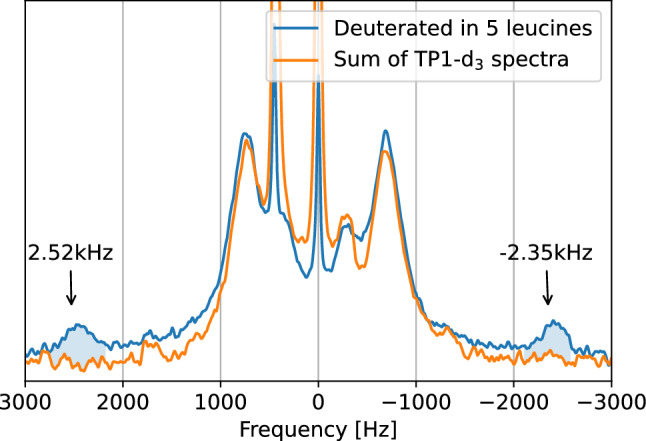
Sum of $^2$H-NMR spectra of membrane-embedded TP1 with deuterium labels in the residues Leu-4, Leu-5, Leu-7 and Leu-8 separately, shown in orange. $^2$H-NMR spectrum of membrane-embedded TP1 with deuterium labels in all five leucine residues simultaneously, shown in blue.

In order to obtain the quadrupolar splitting of the remaining Leu-2 fragment, we compared a sum of the spectra displayed in Fig. 2, with a $^2$H-NMR spectrum of a synthesized TP1 peptide sample with deuterium labels in all five leucine fragments; this comparison is shown in Fig. 3.

From Fig. 3, it can be seen that both profiles are quite similar, except in the shaded area, which is the only signal pair that does not appear in spectra where Leu-4, Leu-5, Leu-7 and Leu-8 are deuterated; therefore, this must be the split signal of the Leu-2 fragment.

**Figure 4:**
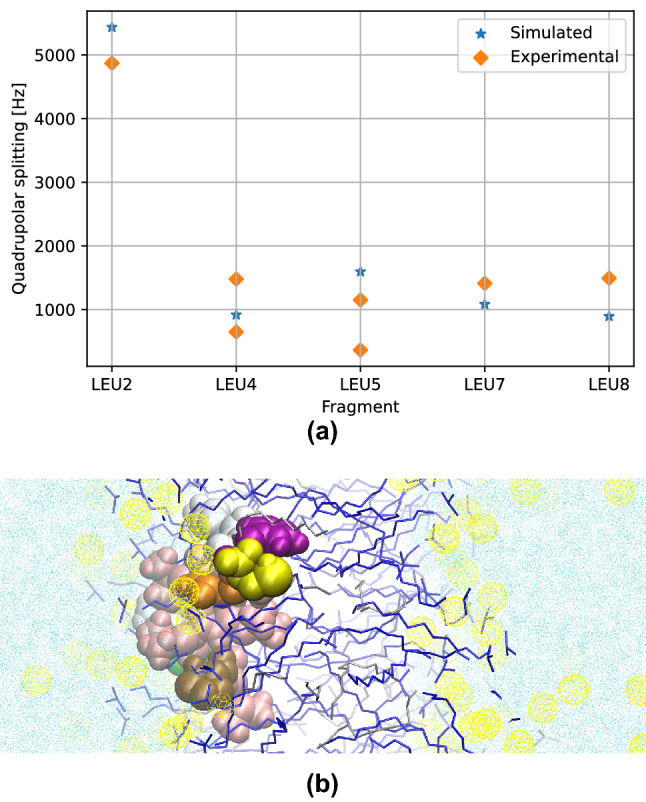
Equilibrium structure of the TP1 peptide as fitted from $^2$H-NMR quadrupolar splittings ($\Delta \nu$) of its leucine fragments. (**a**) Comparison of quadrupolar splittings: simulated and experimental. (**b**) Single frame of the MD simulation of the TP1 peptide at its equilibrium structure when embedded in a membrane mimetic.

In order to associate this measured quadrupolar splitting with an orientation and position of the fragment in the membrane, we performed multiple MD simulations of the peptide embedded in the SDS-based membrane mimetic, each one with the TP1 peptide in different random but stable orientations and membrane depth, until the root-mean-square relative deviation (RMSRD) from the experimental quadrupolar splitting reached a threshold of 50%. After 75 MD simulations, of 40 ns each, a simulation with a RMSRD of 33% was found. A comparison of the quadrupolar splitting obtained from this MD simulation with the experimental quadrupolar splitting is shown in Fig. 4a.

It should be noted, that from the previous 74 iterations, many of the obtained configurations were able to reproduce order parameters and therefore quadrupolar splittings, of few (but not all) of the leucine fragments. And although some of these configurations appeared more often than others, we do not think they have statistical significance, since they could have appeared due to a bias in the initial configuration generation and we only simulated 40 ns per configuration and transmembrane domains of proteins usually stabilize their structure in the range of micro-seconds.

It is interesting to see that the configuration that fits every quadrupolar splitting is in agreement with the preliminary experiment shown in Fig. 1, both cases imply that the TP1 peptide embeds in the outer areas of the membrane bilayer.

**Figure 5:**
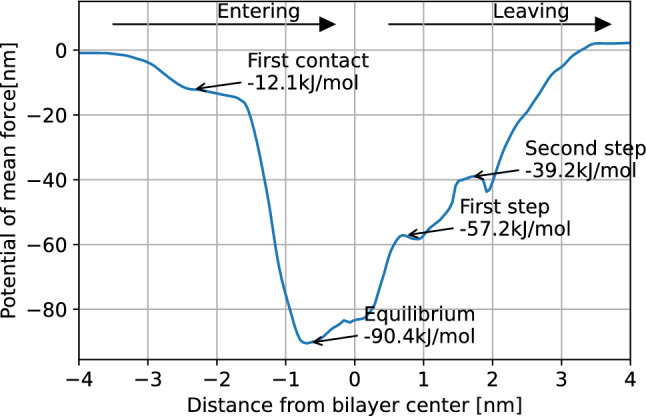
PMF profile of the TP1 peptide translocating a SDS-based membrane mimetic.

With this equilibrium structure, and employing the Umbrella Sampling/WHAM methodology^44^, we calculated a potential of mean force (PMF) profile for the entire process of the TP1 peptide translocating the SDS-based membrane bilayer. A total of 54 NVT simulations, of 40 ns each, were performed to obtain the PMF profile of the full mechanism (shown in Fig. 5).

### Translocation mechanism of the TP1 peptide

**Figure 6:**
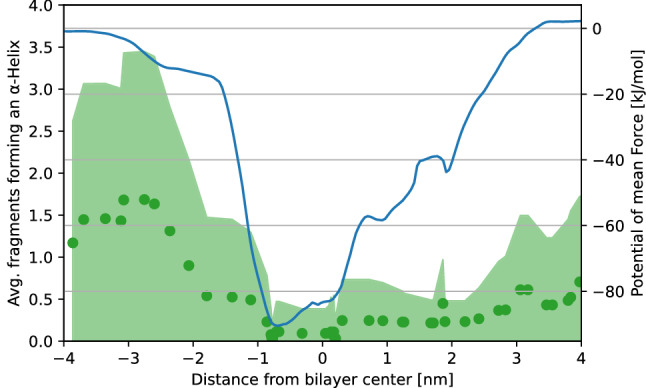
Number of TP1 fragments forming an $\alpha$-Helix structure according to the distance from the bilayer center, time-average values shown as green dots and standard deviation shown as a green shaded area. PMF profile of the same peptide translocating the SDS-based membrane mimetic is shown in a blue line.

**Figure 7:**
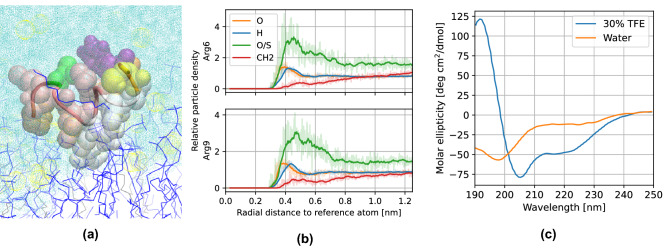
MD simulation of the TP1 peptide penetrating a SDS-based membrane mimetic. (**a**) Single frame of the peptide with its center of mass 2.36 nm away from the bilayer center. Arginine residues are depicted as silver Van der Waals spheres. (**b**) Radial distribution functions of arginine fragments of the peptide upon first interaction with the bilayer. (**c**) Circular dichroism spectra of TP1 peptide in water and 30% TFE solution.

The entire array of simulations performed allowed us to describe a detailed mechanism for the whole process of translocation. During the first interaction of the TP1 peptide with the bilayer, both arginines adsorb onto the outer section of the bilayer (see Fig. 7a), interacting mostly with the (partially) negatively charged sulfate from SDS and oxygen from 1-decanol, as seen from the radial distribution functions of these residues at this stage (see Fig. 7b). Furthermore, the peptide acquires a $\alpha$-helix structure at this stage (see Fig. 6), which coincides with what was found when we performed circular dichroism (CD) studies on the peptide, whereupon adding lipophilic trifluoroethanol (TFE) to the sample, the peptide expressed a CD spectra characteristic of an $\alpha$-helix (see Fig. 7c). The same was observed in simulation: the peptide acquires a $\alpha$-helix structure when exposed to the lipidic components of the bilayer.

After this first contact, the peptide closes up to the bilayer entering an energy potential well (see Fig. 5). This energetic stability is explained by the favorable interactions between the leucine fragments and the aliphatic carbons of the bilayer, as well as the interaction between charged arginine and polar ends of the amphiphilic chains. Being a potential well, the structure we found at this point is the equilibrium structure of the peptide embedded in the bilayer, being the same that we found in previous experiments: the peptide is partially inserted into the membrane, residing mostly in the outer area of the bilayer (see Fig. 4b).

**Figure 8:**
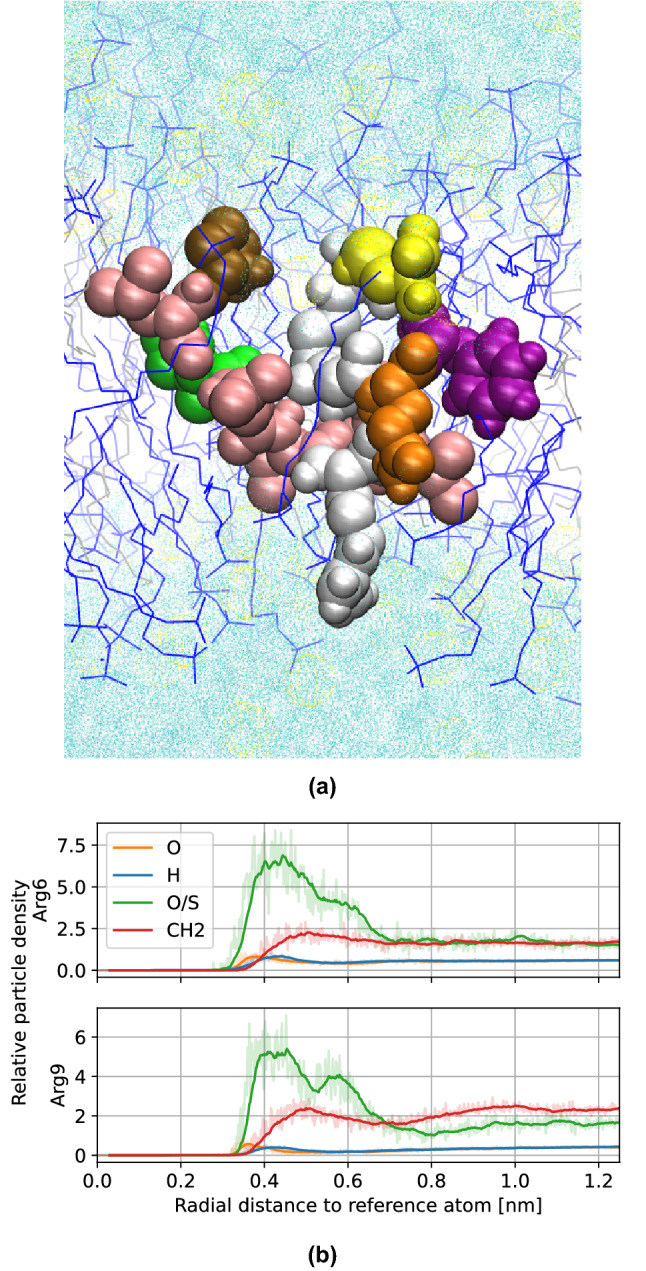
(**a**) Single frame of the TP1 peptide at the first step of the *potential stair*, 0.72 nm away from the bilayer center. (**b**) Radial distribution functions of arginine fragments of the peptide at this stage.

In order to escape the bilayer and translocate the membrane, the peptide must first climb a potential energy *stair*, as seen in the PMF profile. The first step in this *stair* involves arginine-6 reaching the polar heads of the opposite layer of the membrane. The increase in potential energy at this point can be attributed to arginine-9; this fragment which was previously interacting with polar heads of the entrance layer is now close to the center of the bilayer solvated by a few water particles (see Fig. 8).

**Figure 9:**
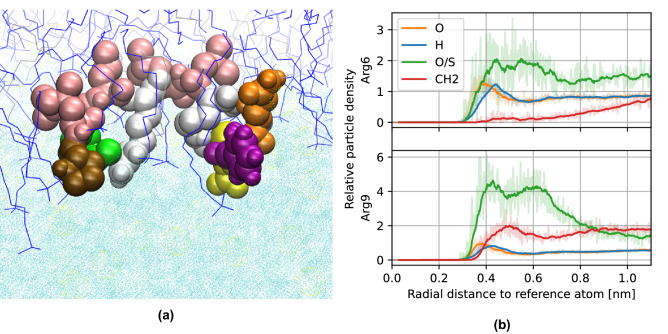
(**a**) Single frame of the TP1 peptide at the second step of the *potential stair*, 1.67 nm away from the bilayer center. (**b**) Radial distribution functions of arginine fragments of the peptide.

Next, the TP1 peptide must climb a second step in this *potential stair*, and is arginine-9 now the one that toggles between layers of the membrane, being found now close to the polar head of the exiting layer of the membrane (see Fig. 9a,b).

Finally, and as a last step, TP1 is released from the exiting layer into the bulk. It should be noted that in our simulation conditions, a peptide that comes into contact with the membrane has the same probability to penetrate as it can back off to where it came from. This is reflected by the same potential value obtained at the beginning as at the end of the translocation (see Fig. 5). This should not be the case in an actual cellular membrane, where the difference of electric potential between the interior and the exterior of the cell, and the peptide concentration gradient both act as a driving force for the translocation of the peptide ensemble. This is to say, in an actual cellular membrane environment, the peptide potential should be lower at the end of the translocation.

## Conclusion

In summary, we developed a novel technique employing the quadrupolar splittings in $^2$H-NMR of a deuterium-labeled peptide along with MD simulations of the same peptide to find its structure while embedded in a membrane mimetic. It should be noted that since we fitted quadrupolar splittings (and therefore order parameters) which depend on the dynamism of the orientation of the deuterium labels, the structure we found is not a static one as is commonly found with crystallographic techniques, but a dynamic one. We believe this technique can be replicated in other transmembrane peptides and protein fragments, and it should be a valuable resource for overcoming the challenge of obtaining the structure and mechanisms of membrane proteins.

From this equilibrium structure, we recreated a possible mechanism for the translocation of the TP1 peptide, where we found that TP1 adsorbs easily into the membrane, but more importantly, it requires a two-step mechanism to translocate the membrane: arginine-6 toggles between layers first, and arginine-9 second. This fact, and considering the structure of the peptide, leads us to believe that the RLLR moiety has special importance in the translocating capabilities of this peptide. Not only are the three amino acids separation between both arginines the ideal distance between each guanidinium group for the two-step translocation mechanism to occur, but also the hydrophobicity of the leucine between these guanidinium groups grants stability to the peptide in the instance where arginine is flip-flopping between layers in the membrane. This conclusion aligns with results found by other researchers^34,35,37^, where higher penetration activity was found in peptides with the RLLR motif.

## Methods

### Membrane mimetic

The reagents sodium dodecyl sulphate (SDS), deuterated sodium dodecyl sulphate (SDS-d$_{25}$), and sodium sulphate ($\hbox {Na}_2\hbox {SO}_4$) were purchased from *Sigma Aldritch*. 1-decanol and HPLC grade water were purchased from *Merck & Co.*. Excepting sodium sulphate which was oven dried for 24 hours before use, all reagents were employed without alterations.

Each SDS-based membrane mimetic sample was prepared by mixing each dry component (SDS and sodium sulphate) in a 5 mL centrifuge tube and then adding 1-decanol and water. Each component proportion was weighted according to the bicelle developed by Bahamondes et al.^41^, which was chosen due to its ability to form magnetically oriented anisotropic liquid crystals. Each sample was then submitted to a rotational mixer spinning at 4 rpm at a temperature of $37\,^\circ \hbox {C}$ until a crystalline sample was obtained after about 24–48 h, and finally centrifuged at 6000 rpm during 3 min.

### Peptide synthesis

Fmoc-protected amino acids and Rink resin were purchased from Iris Biotech, deuterated Fmoc-protected leucine (Fmoc-Leu-OH-5,5,5-d$_3$) was purchased from *Sigma Aldrich*.

TP1 peptide was synthesized without its end-capping cysteine to prevent the formation of dimers. This did not modify its secondary structure, as it did not alter its CD spectrum, and therefore it should not modify its penetrating activity, considering that this cysteine is meant to be disulfide-bonded to a cargo.

TP1 peptide and its deuterated variations were synthesized using standard Fmoc-methodology on Rink amide resin ($0.4\,\hbox {mmol\,g}^{-1}$). The reagents HBTU (N,N,N$^{\prime }$,N$^{\prime }$-tetramethyl-O-(1H-benzotriazol-1-yl) uronium hexafluorophosphate) and HCTU (O-(1H-6-chlorobenzotriazole-1-yl)-1,1,3,3-tetramethyluronium hexafluorophosphate) were used as activators and DMF (N,N-dimethylformamide) was used as solvent. Fmoc deprotections were performed using a solution of 4-methylpiperidine in DMF^45^. Peptide cleavage was carried out with a solution of trifluoroacetic acid, triisopropylsilane, EDT (2,2-(ethylenedioxy)diethanethiol) and water in 92.5:2.5:2.5:2.5 ratio. Reaction products were purified by reverse phase chromatography up to >90% purity^46,47^.

Molecular weight and peptide purity were confirmed by electrospray ionization mass spectroscopy (ESI-MS) and RP-HPLC.

### Nuclear magnetic resonance

All solution $^2$H-NMR experiments were performed on a *Bruker Avance 400* (Universidad de Santiago de Chile, Chile) operating at 61.422 MHz. Spectra were obtained with $90^{\circ }$ $22.4\,\upmu \hbox {s}$ pulses, 760 ms acquisition times and 43.1 KHz spectral width. Spectra were stored in 32 k files with a digital resolution of 1.32 Hz per point.

Spectra involving membrane mimetics were carried out at a temperature of $37\,^{\circ } \hbox {C}$, and a pre-acquisition time of 10 min was employed to allow each sample to reach thermal equilibrium.

Spectra were processed using *NMRGlue* Python library^48^ and analyzed with *MatPlotLib*^49^ and *SciPy*^50^ Python libraries.

### Circular dichroism spectroscopy

CD spectroscopy was carried out on a JASCO J-815 CD Spectrometer (JASCO Corp., Tokyo, Japan) in the far ultra-violet (UV) range (190250), using quartz cuvettes (0.1 cm path length). Each spectrum was recorded averaging three scans in continuous scanning mode. Solvent blank was subtracted from each sample spectrum. Molar ellipticity was calculated for each peptide using $250\,{\upmu }L$ of $2.0\,\hbox {mmol\,L}^{-1}$ peptide in 30% (v/v) 2,2,2-trifluoroethanol and HPLC grade water. Resulting data were analyzed using Spectra Manager software (Version 2.0, JASCO Corp., Tokyo, Japan).

### Molecular dynamics

Molecular dynamics calculations were performed in the National Laboratory for High Performance Computing (NLHPC at Universidad de Chile, Santiago, Chile), as well as in our HPC cluster. All simulations were carried out using GROMACS-2016^51^ software. Periodic boundary conditions were employed in all three dimensions. Single range cut-off scheme of 1.4 ns was employed for non-bonding interactions and long-range electrostatic interactions were calculated with the particle mesh Ewald mehtod^52^. On NVT ensemble simulations, a temperature of $37\,^{\circ }\hbox {C}$ was maintained with a modified Berendsen thermostat^53^, keeping the bilayer and bulk content in separated thermostats, both with a time constant of 1.0 ps. This was done to improve the reproducibility of $^2$H-NMR spectroscopic results^42^. With NPT ensemble simulations, the same temperature schema was kept, while adding pressure control with a semi-isotropic Berendsen barostat^54^, keeping the system at 1 bar in all directions with a time constant of 1.0 ps for both xy-plane and z-axis.

Prior to each production simulation, a transient NPT simulation of 1 ns with an integration time-step of 1 fs was performed to generate velocities and equilibrate the temperature, pressure and energy of the system. Afterwards, each production simulation was performed with an integration timestep of 2 fs.

Initial bilayer configurations were built employing PACKMOL^55^, 40 1-Decanol and 141 SDS molecules were modeled in each $64\,\hbox {nm}^{2}$ layer, this proportion was chosen to reproduce the experimental composition of the SDS-based membrane mimetic (76% SDS and 24% 1-decanol).

PMF calculations were performed using the Umbrella Sampling/WHAM method^44^. The starting configuration of each simulation window was constructed from a trajectory of the peptide pushing through the bilayer and making a starting point each 2.0 Å; further simulations windows were added where the sampling was too poor to represent a reliable PMF, and 57 NVT simulation windows of 40 ns each were calculated in total.

Data analysis of the simulation results was performed using *GromacsWrapper*^56^ and *Pandas*^57^ Python libraries. Secondary structure analysis was performed using *DSSP* software^58^.

## Data Availability

All the simulation raw data, including the equilibrium structure mentioned, are available at our laboratory repository following the link: https://sauron.ciencias.uchile.cl/gitea/diego-m/TP1_en_bicapa_SDS.
